# Identification of rare variants for hypertension with incorporation of linkage information

**DOI:** 10.1186/1753-6561-8-S1-S109

**Published:** 2014-06-17

**Authors:** Yen-Feng Chiu, Ren-Hua Chung, Chun-Yi Lee, Hui-Yi Kao, Lin Hou, Fang-Chi Hsu

**Affiliations:** 1 Division of Biostatistics and Bioinformatics, Institute of Population Health Sciences, National Health Research Institutes, 35 Keyan Rd., Zhunan, Miaoli 35053, Taiwan, ROC; 2 Department of Biostatistics, Yale School of Public Health, 60 College Street, New Haven, Connecticut 06520, USA; 3 Department of Biostatistical Sciences, Division of Public Health Sciences, 1834 Wake Forest Rd., Wake Forest School of Medicine, Winston-Salem, NC 27157, USA

## Abstract

We conducted linkage analysis using the genome-wide association study data on chromosome 3, and then assessed association between hypertension and rare variants of genes located in the regions showing evidence of linkage. The rare variants were collapsed if their minor allele frequencies were less than or equal to the thresholds: 0.01, 0.03, or 0.05. In the collapsing process, they were either unweighted or weighted by the nonparametric linkage log of odds scores in 2 different schemes: exponential weighting and cumulative weighting. Logistic regression models using the generalized estimating equations approach were used to assess association between the collapsed rare variants and hypertension adjusting for age and gender. Evidence of association from the weighted and unweighted collapsing schemes with minor allele frequencies ≤0.01, after accounting for multiple testing, was found for genes *DOCK3 *(p = 0.0090), *ARMC8 *(p = 1.29E-5), *KCNAB1 *(p = 5.8E-4), and *MYRIP *(p = 5.79E-6). *DOCK3 *and *MYRIP *are newly discovered. Incorporating linkage scores as weights was found to help identify rare causal variants with a large effect size.

## Background

Linkage studies have high power to detect loci that have variants with large effect size, although they often are rare in the population [1]. In contrast, association studies generally have high power to detect common variants with a small effect size for diseases or traits [2]. Recently, next-generation sequencing techniques have made feasible sequencing of all exons or the whole genome of an adequate number of individuals for meaningful results. Rare variant analysis is challenging because of sequencing-based uncertainties in variant calling, the large search space of rare variants, and the inherently low carrier rate frequencies. Rare variants, however, are quite common in the general population. Therefore, it could be helpful to apply linkage analysis on these new DNA sequencing data to identify rare causal variants with a large effect size [3]. In the present study, we conducted linkage analysis using genome-wide association studies (GWAS) data to identify disease susceptibility loci, then applied logistic regression models using generalized estimating equations (GEEs) to assess the associations between hypertension and rare variants of the susceptibility genes in the linked regions.

## Methods

### GWAS and phenotype data

Linkage analysis was conducted on chromosome 3 GWAS data. A total of 65,519 single-nucleotide polymorphisms (SNPs) were genotyped on chromosome 3 for 959 individuals from 20 original pedigrees; of these individuals, 344 had hypertension and 506 did not. As a result of the limitations of our computing facility for linkage analysis, PedCut [4] was used to split large pedigrees with members greater than 20 bits into smaller pedigrees to enable analyses by MERLIN [5]. Consequently, we analyzed a total of 138 pedigrees with 1495 individuals (missing parents were added in); for the divided pedigrees, pedigrees ranged from 3 to 25 individuals. Five SNPs were removed for failing the Hardy-Weinberg equilibrium (*p *value < 10^-4^) test. The Hardy-Weinberg equilibrium test was performed using PLINK 1.07 [6] based on 56 unrelated subjects. A total of 22,056 genotypes with genotyping errors (genotyping error rate was approximately 3.51 × 10^−4^) were further excluded by the MERLIN 1.1.2 computing package [5]. Subjects being diagnosed with hypertension for at least 1 of the 4 time points were considered as affected.

### Linkage and association analysis

Linkage screens on chromosome 3 GWAS data were conducted using MERLIN 1.1.2; linkage evidence was assessed based on nonparametric linkage (NPL) log of odds (LOD) scores by Kong and Cox [7] where identity-by-descent sharing in affected relative pairs was computed. Because of the heavy computational load from the tremendous number of markers, linkage analyses were performed with an interval of 1000 SNPs in 1 run. Each interval had a 5-SNP duplicate with its following interval. One-LOD support intervals were constructed for each linkage peak with NPL LOD scores ≥4.0. Genes located in the 1-LOD support intervals were identified and annotated based on the genetic map NCBI build 36. Rare variants--defined as variants with minor allele frequency (MAF) ≤0.01, 0.03, or 0.05--in each gene were collapsed, either unweighted or weighted by the LOD scores, in 2 ways: exponential weighting and cumulative weighting [8]. Namely, a rare variant *i *with LOD score *z_i _*was weighted by $w_{i}=\upsilon_{i}/{\bar{\upsilon }}_{m}$ if a subject carried at least 1 minor allele of SNP *i *and by, otherwise. Here, $\upsilon_{i}=e^{Z_{i}}$ for the exponential weighting, $\upsilon_{i}=\Phi \left(z_{i}-2\right)$ for the cumulative weighting, Φ(⋅) is the standard normal cumulative distribution, and ${\bar{\upsilon }}_{m}=\frac{1}{m}\overset{m}{\underset{i=1}{\sum }}\upsilon_{i},$ where *m *is the total number of rare variants per gene, *i *= 1,..., *m*. For individual *k*, his or her collapsed rare variants (CRVs) were then equal to $\overset{m_{k}}{\underset{i=1}{\sum }}w_{i},$ assuming the total number of rare variants the individual carries was *m_k_*. The association between hypertension and the CRVs was assessed by logistic regression models adjusted for age and gender. The GEE approach implemented in the SAS computing package (SAS Inc., Cary, NC) was used to account for within-family correlations in the association analysis based on the original 20 families under an exchangeable covariance structure. Multiple testing corrections were made using the false discovery rate (FDR) as implemented in SAS.

## Results

**Figure 1:**
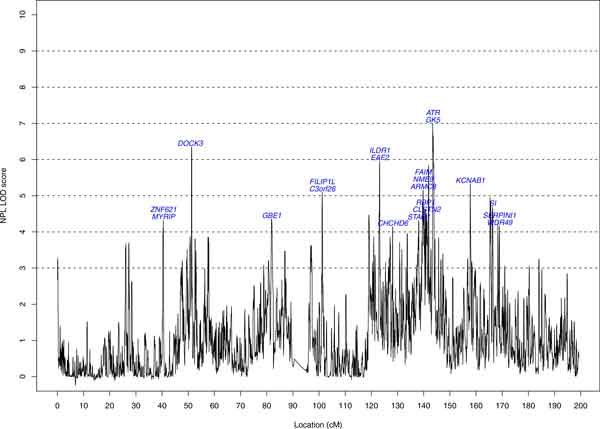
**NPL LOD score on chromosome 3 of GWAS data**. *cM*, centimorgan.

Figure 1 displays the NPL LOD scores for chromosome 3 GWAS data. The highest peak is located at 143.428 megabases (Mb) with a NPL LOD score of 7.0. Thirteen support intervals with NPL LOD scores ≥4 and the genes located in the 1-LOD support intervals were identified (Table 1). A total of 21 genes are harbored in these regions. Fifteen (71%) of these 21 genes were identified in previous linkage analyses for quantitative blood pressure (Table 1) [9]. As the GWAS map was denser than the maps in previous linkage studies, the information content was richer; more regions were identified and their support intervals were more narrowly based on the current denser markers [10]. Table 1 also lists the estimates of CRVs for individual genes with 3 weighting schemes under MAF ≤0.01. The results for MAF ≤0.03 and MAF ≤0.05 are not shown. The most striking association was with the gene *ZNF621 *(estimated effect −0.44, *p *<1.0E-30). The effect is from the single variant, rs34412695, with MAF = 0.00096. This association should be interpreted with caution because the significance resulted from only 1 rare variant with 1 minor allele. A larger sample size is required to reexamine this finding. The risk variant(s) in gene *ATR *(LOD score = 7.0) had MAFs between 0.01 and 0.03, hence the CRV is significant (*p *= 0.024) for the MAF ≤0.03 category only. The CRV for gene *DOCK*3 has a risk effect (*p *= 0.0090) comprised of 51 variants with MAF ≤0.01. The significance was reduced after collapsing with other variants (*p *= 0.043 for the MAF ≤0.03 category and *p *= 0.056 for the MAF ≤0.05 category). The CRV for the *ARMC8 *gene (with 6 variants collapsed) had a significant protective effect (*p *= 2.27E-5). The CRV (with 16 MAF ≤0.01 variants combined) for the *KCNAB*1 gene had a significant positive effect (*p *= 0.00058); the significance is reduced when collapsing with more variants (*p *= 0.048 and 0.15 in the MAF ≤0.03 and MAF ≤0.05 categories, respectively). The CRV of the *MYRIP *gene in the MAF ≤0.01 category had a significant protective effect (*p *= 1.39E-5), which was not present in the other 2 categories. In the present study, the power to detect the collapsed causal variants with a large effect size from *ARMC8 *and *MYRIP *genes, was improved when incorporating linkage scores as weights based on the GEE approach. We did not observe an improvement in power for the collapsed variants with small effect sizes. Regardless, the *ZNF621 *(FDR <1E-30)*, DOCK3 *(FDR = 0.031), *ARMC8 *(FDR = 0.00013), *KCNAB1 *(FDR = 0.0025), and *MYRIP *(FDR = 0.00012) genes remained significant after a multiple testing correction, regardless of the weighting schemes--the FDRs provided are for the unweighted CRV.

**Table 1:** Estimates for the effects of the CRVs in individual genes with 3 weighting schemes for variants with MAF ≤0.01

		Without weighting			Exponential weighting			Cumulative weighting			
			
Gene*	LOD score	Estimate (SE)	*p *Value	FDR	Estimate (SE)	*p *Value	FDR	Estimate (SE)	*p *Value	FDR	Previous hits†
*GK5 *(1)	7.00	−0.013 (0.023)	0.57	0.75	−0.013 (0.023)	0.57	0.69	−0.013 (0.023)	0.57	0.75	L
*ATR *(10)	7.00	0.055 (0.038)	0.15	0.28	0.057 (0.036)	0.12	0.23	0.055 (0.038)	0.15	0.28	L

*DOCK3 *(51)	3.87-6.35	0.035 (0.013)	0.0090	0.031	0.0089 (0.0043)	0.040	0.12	0.025 (0.0098)	0.013	0.044	

*C3orf26 *(24)	5.09	0.023 (0.046)	0.62	0.75	−0.050 (0.056)	0.37	0.57	0.022 (0.048)	0.64	0.78	L
*FILIP1L *(20)	5.09	−0.048 (0.053)	0.37	0.57	−0.092 (0.065)	0.16	0.27	−0.049 (0.058)	0.40	0.62	L

*ILDR1 *(1)	5.96	−0.032 (0.12)	0.79	0.84	−0.032 (0.12)	0.79	0.90	−0.032 (0.12)	0.79	0.86	

*ARMC8 *(6)	5.16	−0.13 (0.031)	2.27E-5	0.00013	−0.12 (0.028)	1.29E-5	7.31E-5	−0.13 (0.031)	2.24E-5	0.00013	L
*NME9 *(5)	5.16	−0.085 (0.049)	0.081	0.20	−0.083 (0.048)	0.081	0.20	−0.085 (0.049)	0.081	0.20	L
*FAIM *(2)	5.16	−0.13 (0.090)	0.14	0.28	−0.14 (0.089)	0.11	0.23	−0.13 (0.090)	0.14	0.28	L

*KCNAB1 *(16)	3.05-5.33	0.054 (0.016)	0.00058	0.0025	0.065 (0.020)	0.0011	0.0047	0.060 (0.018)	0.00077	0.0033	LG

*MYRIP *(4)	4.32	−0.22 (0.050)	1.39E-5	0.00012	−0.20 (0.044)	6.29E-6	5.35E-5	−0.19 (0.042)	5.79E-6	4.92E-5	
*ZNF621 *(1)	4.32	−0.44 (0.021)	<1.0E-30	<1.0E-30	−0.44 (0.021)	<1.0E-30	<1.0E-30	−0.44 (0.021)	<1.0E-30	<1.0E-30	

*GBE1 *(2)	4.36	−0.32 (0.16)	0.039	0.11	−0.32 (0.16)	0.041	0.12	−0.32 (0.16)	0.039	0.11	LG

*CHCHD6 *(24)	4.14	0.00037 (0.013)	0.98	0.98	0.0018 (0.011)	0.88	0.90	0.00093 (0.013)	0.95	0.95	G

*STAG1 *(23)	4.32	0.011 (0.042)	0.79	0.84	0.0047 (0.037)	0.90	0.90	0.010 (0.041)	0.81	0.86	L

*CLSTN2 *(20)	4.52	0.027 (0.027)	0.30	0.51	0.015 (0.023)	0.51	0.69	0.026 (0.026)	0.32	0.54	L

*WDR49 *(11)	4.16	0.043 (0.063)	0.49	0.69	0.024 (0.043)	0.57	0.69	0.031 (0.046)	0.50	0.71	L

*Numbers in parentheses are the total numbers of rare variants being collapsed in each gene. *SERPINI1 *genes were excluded from the list for not having any SNPs with MAF ≤0.01.

†Linkage (L) and/or GWAS (G) hits for quantitative blood pressure in the previous studies.

## Discussion

Linkage scores from GWAS data can be useful to narrow down regions for detecting rare variants associated with disease. Therefore, using linkage scores as weights for collapsing rare variants may improve the power of detection. Although in the present study, the effects of rare variants were assumed to be in the same direction during collapsing, it is important to take the directions of effects into consideration during collapsing, as the effects of significant variants can be diluted or eliminated when collapsed with other variants having neutral or opposite effects. One way to eliminate this problem is to test the variation of individual variant effects, rather than their mean effects, in mixed-effects models [11]. Studying CRVs from different collapsing categories helped identify the MAF category yielding consistent results over genes, because the significance of CRV depends on the thresholds for collapsing. Intuitively, a variant may be functional if its MAF is below a certain threshold; therefore, a varying-threshold approach has proved to be helpful with the identification of functional variants [12]. Incorporating a varying-threshold approach may improve power to detect functional rare variants. In general, the changes in effect sizes resulting from collapsing additional variants or weighting decreased as MAF thresholds increased. Collapsing additional variants often reduced the effect size (results not shown), whereas weighting usually increased the effect size, particularly when the MAF threshold was small. In addition, the significance of *ZNF621 *with an effect size of −0.44 (SE = 0.021) under MAF ≤0.01 resulted from only a single allele. The effect size changed to 0.045 (SE = 0.057) and became insignificant after collapsing with the other 2 variants under MAF ≤0.03 or ≤0.05. This observation suggested the necessity to carefully reexamine and interpret the significant result that was based on only a few rare variants.

Accounting for multiple testing, the CRVs from the following 4 genes were identified for hypertension: *DOCK3, ARMC8, KCNAB1*, and *MYRIP. KCNAB1 *was the only gene previously identified in a GWAS, specifically for being associated with blood pressure. The other 3 genes were novel for hypertension/blood pressure in the present association analysis. Our proposed method focused on rare variant detection; common variants were not analyzed in the association analyses. Therefore, we did not expect to have a large proportion of replicate findings from GWAS.

The 20 families varied in size and ranged from 22 to 86 individuals, so it may not be reasonable to use an exchangeable correlation structure in the GEE approach. However, independent and exchangeable correlation structures involving less covariance parameters were better options than others given such a small number of families. An exchangeable correlation structure was adopted here as it is often a more appropriate correlation structure for a family study than other structures [13]. GEE approaches have robust variance estimators for extended pedigrees in a genome-wide association study setting [14,15]. However, because of the limited sample size in the present study, some of the collapsed variants were not as robust as the others (data not shown). After applying an independent correlation structure, we observed that the *KCNAB1 *gene became insignificant, whereas the genes *GBE1 *and *GK5 *were significant under the unweighted scheme, accounting for multiple testing. In such conditions, it may be helpful to apply a statistical method to select an appropriate variance-covariance structure [16]. This possibility will be investigated in a future study.

## Conclusion

In summary, it is helpful to apply linkage analysis to GWAS or sequencing data, and then incorporate the linkage information into association analyses under certain scenarios. The benefits of using this method were seen particularly in cases where the collapsed variant had a large effect size. A powerful collapsing method should consider the effect size and direction of a rare variant, as well as the threshold of MAF during collapsing. We are currently systematically studying and modifying this proposed method under different scenarios to improve its power to detect functional rare variants.

## Competing interests

The authors declare that they have no competing interests.

## Authors' contributions

YFC, RHC, LH, and FCH made contributions to the study design, statistical analysis, interpretation, and draft of the manuscript. HYK and CYL performed the data analysis. All authors read and approved the final manuscript.
